# Cirrhosis and liver transplantation in patients co-infected with HIV and hepatitis B or C: an observational cohort study

**DOI:** 10.1007/s15010-016-0976-x

**Published:** 2017-01-04

**Authors:** Charlotte Warren-Gash, Kate Childs, Alicia Thornton, Sanjay Bhagani, Shirin Demma, Ankur Srivastava, Clifford Leen, Kosh Agarwal, Alison J. Rodger, Caroline A. Sabin, On behalf of the Joint UK CHIC and liver transplant advisory group

**Affiliations:** 1 Institute of Health Informatics, University College London, London, UK; 2 Institute of Liver Studies, King’s College Hospital, London, UK; 3 Research Department of Infection and Population Health, University College London, London, UK; 4 Department of Infectious Diseases, Royal Free London NHS Foundation Trust, London, UK; 5 Institute for Liver and Digestive Health, Division of Medicine, University College London, London, UK; 6 Hepatology Unit, Department of Experimental and Clinical Medicine, University of Catania, Catania, Italy; 7 Regional Infectious Diseases Unit, Western General Hospital, Edinburgh, UK; 8 The Farr Institute of Health Informatics Research, 222 Euston Road, London, NW1 2DA UK

**Keywords:** HIV, Hepatitis B, Hepatitis C, Co-infection, Cirrhosis, Liver transplantation

## Abstract

This study assessed the likelihood of referral for liver transplantation assessment in a prospective cohort of patients co-infected with HIV and hepatitis B or C with complications of cirrhosis. There were 141 co-infected patients from 11 UK centres with at least one complication of cirrhosis recorded (either decompensation or hepatocellular carcinoma) out of 772 identified with cirrhosis and/or HCC. Only 23 of these 141 (16.3%) were referred for liver transplantation assessment, even though referral is recommended for co-infected patients after the first decompensation episode.

## Introduction

Chronic hepatitis C and hepatitis B infections are more common in people with HIV than those without HIV [1]. Co-infection with HIV and viral hepatitis increases both the likelihood and rate of progression to cirrhosis, hepatocellular carcinoma and end-stage liver disease compared to either HIV infection or hepatitis mono-infection alone [2, 3]. Liver disease is a leading cause of death in HIV-positive individuals [1], and is thus responsible for a considerable mortality burden particularly in countries where widespread access to combination antiretroviral therapy (cART) has drastically reduced numbers of deaths from AIDS.

Historically, liver transplantation—an important treatment option for patients with end-stage liver disease—has been successful in the context of HBV/HIV co-infection [4] but has carried poor outcomes in HIV/HCV co-infected patients. This was principally due to poor patient and graft survival as the immunosuppression required to prevent organ rejection frequently resulted in infectious complications as well as aggressive HCV recurrence [5]. Now, as well as improved survival in HIV-positive persons due to cART, recent advances in the treatment of hepatitis C with directly acting antiviral agents (DAAs) and prophylaxis of opportunistic infections mean that liver transplantation may be an increasingly viable option for co-infected patients with end-stage liver disease [6].

Recent clinical practice around liver transplantation in co-infected patients in the United Kingdom, however, remains unclear. Here we aimed to describe the likelihood of referral for liver transplantation assessment in a cohort of co-infected patients with complications of cirrhosis seen from 2004 onwards, using linked routine data from multiple UK centres.

## Methods

### Study design, setting and participants

A cohort of co-infected patients with cirrhosis was drawn from a sub-study of 11 centres from the United Kingdom Collaborative HIV Cohort (UK CHIC) study—a multi-centre prospective cohort study collecting routine clinical and treatment data on HIV-positive individuals [7]. Pseudonymised data are collected annually from all participating centres. UK CHIC data are also updated each year using linked HIV surveillance datasets from Public Health England, which are linked to national death registry data. Individuals in the hepatitis sub-study had evidence of either hepatitis B, defined as ever receiving a positive hepatitis B surface antigen or positive HBV-DNA test, or hepatitis C infection, defined as ever receiving a positive hepatitis C antibody test or a positive hepatitis C RNA test, and had received care at one of 11 tertiary centres in England and Scotland from 2004 onwards. Cirrhosis was defined as either a confirmed diagnosis (APRI score >2; biopsy with Ishak score of 5 or 6; biopsy with METAVIR score = 4; FibroScan with result >14 kPa), or a scan result suggestive of cirrhosis. We also included data on co-infected patients with hepatocellular carcinoma (HCC). Supplementary data were collected on clinical outcomes of co-infection including decompensation events identified through medical records review between September 2012 and September 2013 at the included centres. The UK CHIC dataset was enriched by collecting additional data on liver transplant assessments and outcomes in co-infected patients occurring up to December 2014 from three major liver transplant centres: King’s Liver Transplant Unit, the Sheila Sherlock Liver Centre at the Royal Free London NHS Foundation Trust and the Scottish Liver Transplant Unit based at the Royal Infirmary of Edinburgh. These centres are responsible for 44% of liver transplants carried out in the UK [8] and cover similar geographical areas to the HIV treatment centres contributing data to the UK CHIC study. This methodology was previously used to investigate outcomes of end-stage kidney disease in UK CHIC participants [9].

### Data linkage

Data from liver transplant units were linked to data from the UK CHIC hepatitis sub-study using a deterministic n-1 approach with the matching variables day of birth, month of birth, year of birth, gender, hepatitis B status and hepatitis C status, i.e., a match was considered true if at least five of these six variables matched. In addition, true matches could not have a death date recorded in either UK CHIC or transplant data before the end of follow-up in the other dataset.

This approach allowed for minor data entry errors, missing data fields to be present and time lags between the two datasets in which hepatitis B or C recorded status could change with serological resolution of infection or re-infection/reactivation. A total of 48 records from transplant units were collected, of which 34 (70.8%) matched to a UK CHIC record. Of the 34 matched records, 14 (41.2%) matched on all 6 fields and 20 (58.8%) matched on 5 fields but were considered likely to be true matches by manual record review.

### Statistical analysis

We described the proportion of co-infected patients with cirrhosis experiencing complications (either decompensation or HCC), the proportion referred for transplant assessment and receiving a transplant stratified by the presence or absence of complications, and the proportion that died during follow-up in UK CHIC. *χ* ^2^ tests were used to compare outcomes by clinical or demographic variables such as gender, age group and infection status. We also described characteristics of those referred for transplant including stages of liver disease using UKELD [10], MELD and Child Pugh scoring systems, and assessed differences in those with repeated measures at assessment and transplant using paired *t* tests. We did not undertake further analysis on any sub-group containing fewer than 10 individuals to reduce the risk of any individual being identified, as per research ethics committee guidance. All analyses were conducted using Stata version 13 (StataCorp LP, College Station, TX).

## Results

Of 4659 people co-infected with hepatitis B or C in the UK CHIC study, 575 (12.3%) had confirmed cirrhosis, a further 190 (4.1%) had imaging suggestive of cirrhosis and 7 (0.15%) had HCC but no cirrhosis record. For these 772 patients, 141 (18.3%) had at least one complication of cirrhosis recorded, rising to 29.2% in triple infected patients. For the 141 patients with a complication of cirrhosis recorded, median age was 44.6 years (IQR 40.1–49.3 years), 83.7% were male and median follow-up time was 13.1 years (IQR 6.2–18.7 years). Demographic and clinical characteristics stratified by infection status are shown in Table 1 for the cirrhosis cohort with and without recorded complications.

**Table 1:** Characteristics of 772 co-infected patients with cirrhosis or HCC in the UK CHIC Study

Characteristic	HIV/hepatitis B, *n* (%)	HIV/hepatitis C, *n* (%)	HIV/hepatitis B and C, *n* (%)	Total, *n* (%)
Gender				
Male	177 (86.3)	400 (83.7)	80 (89.9)	657 (85.1)
Female	28 (13.7)	78 (16.3)	9 (10.1)	115 (14.9)
Age at cirrhosis/HCC diagnosis (years)				
<30	9 (4.4)	32 (6.7)	4 (4.5)	45 (5.8)
30–34.9	28 (13.7)	54 (11.3)	16 (18.0)	98 (12.7)
35–39.9	33 (16.1)	93 (19.5)	16 (18.0)	142 (18.4)
40–44.9	48 (23.4)	119 (24.9)	20 (22.5)	187 (24.2)
45–49.9	47 (22.9)	98 (20.5)	17 (19.1)	162 (21.0)
50–54.9	21 (10.2)	47 (9.8)	10 (11.2)	78 (10.1)
55+	19 (9.3)	35 (7.3)	6 (6.7)	60 (7.8)
Method of cirrhosis diagnosis (*n* = 765)^a^				
APRI score	71 (35.3)	289 (60.5)	27 (31.4)	387 (50.6)
Liver biopsy	32 (15.9)	60 (12.6)	25 (29.1)	117 (15.3)
Fibroscan	12 (6.0)	44 (9.2)	15 (17.4)	71 (9.3)
Imaging suggestive of cirrhosis	86 (42.8)	85 (17.8)	19 (22.1)	190 (24.8)
Complication (at least one episode)				
Ascites	16 (7.7)	36 (7.5)	10 (11.2)	62 (8.0)
Portal hypertension	28 (13.6)	50 (10.5)	16 (18.0)	94 (12.2)
Varices ± haematemesis	30 (14.6)	52 (10.9)	18 (20.2)	100 (12.9)
Encephalopathy	^b^	^b^	^b^	6 (0.8)
Hepatocellular carcinoma	17 (8.3)	8 (1.7)	5 (5.6)	30 (3.9)
Any of the above	46 (22.3)	69 (14.4)	26 (29.2)	141 (18.2)
Death				
Yes	33 (16.1)	73 (15.3)	13 (14.6)	119 (15.4)
No	172 (83.9)	405 (84.7)	76 (85.4)	653 (84.6)
Median time to death from cirrhosis or HCC diagnosis (IQR) (years)	3.4 (0.6–6.8)	3.2 (0.7–7.1)	3.1 (1.2–5.1)	3.3 (0.7–6.7)
Total	205 (26.6)	478 (61.9)	89 (11.5)	772 (100.0)

^a^ *n* = 765 for method of cirrhosis diagnosis. An additional seven patients had evidence of HCC alone

^b^Suppressed due to small numbers

For patients with a complication recorded, the proportion referred for liver transplantation assessment was 23/141 (16.3%). The corresponding proportion for patients with no record of complications (who may not meet referral criteria) was 15/631 (2.4%). Of these 38 patients with evidence of referral for transplant assessment, this was based on either transplant unit data alone (*n* = 27), transplant and UK CHIC data (*n* = 6) or UK CHIC data denoting a previous transplant (*n* = 5). The proportions receiving a transplant assessment did not differ significantly by gender (*p* = 0.09), age group (*p* = 0.67) or infection status (*p* = 0.31). Although numbers were small, there was some evidence that referrals increased over time: there were 8 referrals between 2003 and 2006, 10 referrals between 2007 and 2010 and 14 referrals between 2011 and 2014; a further 6 had no recorded transplant assessment date. Of patients with transplant unit data who were assessed, 22 (66.7%) were accepted onto the transplant list, three were deferred (9.1%) and eight (24.2%) were not accepted. Reasons given for not accepting patients onto the transplant list included being too well, psychosocial reasons such as ongoing drug or alcohol use, and alternative management decisions, e.g., tumour resection for some patients with HCC. Four patients on the list were delisted before transplant for similar reasons.

There were 25 patients with recorded cirrhosis or HCC who had evidence of receiving a liver transplant (14 from transplant unit data alone, six from transplant and UK CHIC data and five from UK CHIC data alone), which included some patients initially deferred or not accepted onto the list at first assessment. Among those assessed, receiving a transplant was not associated with age group (*p* = 0.90), gender (*p* = 0.75) or infection status (*p* = 0.99). Where data were available from transplant units (20 individuals), the median waiting time from date of referral for assessment to transplant was 6.7 months (IQR 4.5–11.2). For these 20 patients, 25% had evidence of at least one episode of decompensation while on the transplant waiting list, 50% had no episodes, with no data on decompensation available for the remaining 25%. Characteristics of patients at the time of assessment and transplant are shown in Table 2.

**Table 2:** Characteristics of patients assessed for and receiving liver transplants

Characteristic	Referred for transplant (*n* = 33), median (IQR)	Had a transplant (*n* = 20)^a^, median (IQR)	*p* value^±^
UKELD score	55 (51–63)	58.5 (48–66.3)	0.69
MELD score	14 (10–21)	14 (9–27)	0.34
Child Pugh score	9 (6.5–10.5)	9.5 (6–12)	0.45
Body mass index	–	23.5 (21.6–26.0)	–
ICU duration (days)	–	3.5 (2–9)	–
Total duration of stay (days)	–	20.5 (15.8–30.5)	–

^a^An additional five patients with CHIC data on transplants did not have information on stage of liver disease by UKELD, MELD or Child Pugh score, or details of BMI or length of hospital stay

^±^From paired *t* test for individuals with measures at both assessment and transplant

There were 57 deaths among people with a complication recorded at any time during follow-up (40.4%) compared to 62 deaths among those with no complications recorded (9.8%). Results were similar when stratifying by transplant status: 42.9% with a complication died versus 0% without a complication among patients with a transplant recorded; corresponding results for those with no transplant record were 40.2 versus 10%, respectively. There were no significant differences in the proportion who died between those who did and did not have a record of transplant (*p* = 0.23). The proportion of patients who died also did not differ by gender (*p* = 0.53), age at cirrhosis/HCC diagnosis (*p* = 0.47) or infection status (*p* = 0.94). It was not possible, however, to investigate survival in detail due to differences in follow-up time between those with and without transplant data.

## Discussion

In our cohort of co-infected patients with complications of cirrhosis, the proportion referred for liver transplantation assessment was low (16.3%), even after enriching the dataset with additional data from transplant centres. Survival of HIV/HCV co-infected patients with decompensated cirrhosis without liver transplantation was extremely poor in the pre-DAA era (median = 16 months after first decompensation episode) [11]. Current British HIV Association guidelines recommend that patients with HIV/viral hepatitis co-infection with cirrhosis should be referred for transplantation assessment early, and no later than after first decompensation [12]. Our work highlights the need to optimise current management of end-stage liver disease in this patient population.

The reasons for low rates of referrals for transplant assessment in patients with HIV/HBV coinfection are not clear, given that transplant results are excellent, with no difference in patient and graft survival between HBV mono-infected and HBV/HIV co-infected individuals [4]. In contrast, it is possible that concerns over poor liver transplant outcome in patients with HIV/HCV co-infection may have contributed to clinician reluctance to refer. In studies prior to the advent of DAAs, these individuals experienced lower patient and graft survival and higher rates of acute rejection than patients with HCV mono-infection [13–15]. In HIV/HCV co-infected patients post-transplant, HCV recurrence is the main cause of death [14, 15] and clearance of HCV virus dramatically improves survival [15].

Outcomes in HIV/HCV are likely to improve in the DAA era such that patients with HIV/HCV should be able to achieve an HCV cure either before a transplant is necessary or post-transplant. Clinical trials of DAAs show excellent sustained virologic response (SVR) rates in HIV/HCV co-infected patients across all genotypes that are similar or better than those seen in HCV mono-infected patients [16, 17]. There is also evidence that treating HCV with DAAs in the post-transplant setting in HCV mono-infection can achieve SVR in the majority of patients [18].

Strengths of our study include drawing on a large HIV patient cohort from multiple centres around the UK with detailed long-term follow-up on liver disease diagnoses and management. Collecting additional data from three major liver transplant units improved ascertainment of referrals for transplant assessments. The transplant units covered similar geographical areas to the HIV treatment centres contributing data to the UK CHIC study so it is likely that most patients in the CHIC study referred for liver transplantation assessment would have been referred to one of these units. This method of linking data from transplant units to UK CHIC data has previously been used successfully to investigate rates of kidney transplantation in the context of HIV infection [9].

There are, however, several limitations to our study. The specificity of a cirrhosis diagnosis varied depending on the method used: we recognise that the APRI score has less diagnostic accuracy in co-infected patients compared to mono-infected patients, and some patients were included based on imaging suggestive of cirrhosis. We adopted an intentionally broad definition for inclusivity, but recognised that not all would meet criteria for liver transplantation. Lack of data on other parameters such as MELD score in patients without linked transplant unit data meant that the exact number eligible for transplant was uncertain. Nevertheless, the proportion referred for transplant assessment was low when restricting to patients with evidence of decompensation. This limited statistical power to detect differences in outcomes by factors such as co-infection status. There may also have been incomplete recording of decompensation events in UK CHIC data: previous studies have shown varying rates of decompensation among HCV/HIV co-infected individuals ranging from 5.8% among all co-infected individuals [19] to 23% among cirrhotic co-infected individuals [20], but varying lengths of follow-up make these difficult to compare across studies. Incomplete recording of decompensation in UK CHIC data would, however, artificially increase rather than decrease the proportion referred for liver transplantation assessment. Finally, differing lengths of follow-up time between patients with UK CHIC data alone and those who had additional transplant unit data limited the scope for conducting survival analysis.

In conclusion, we show that management of decompensated cirrhosis in co-infected individuals in the UK is sub-optimal due to the low proportions of patients referred for and receiving liver transplantation. The recent revolution in antiviral treatments for hepatitis C means that clinicians must ensure that co-infected patients have the opportunity for early transplant assessment to facilitate optimal and timely management of their end-stage liver disease.
